# Serological and cellular inflammatory signatures in end‐stage kidney disease and latent tuberculosis

**DOI:** 10.1002/cti2.1355

**Published:** 2021-11-05

**Authors:** Milla R McLean, Kathleen M Wragg, Ester Lopez, Sandra A Kiazyk, Terry Blake Ball, Joe Bueti, Stephen J Kent, Jennifer A Juno, Amy W Chung

**Affiliations:** ^1^ Department of Microbiology and Immunology University of Melbourne at The Peter Doherty Institute for Infection and Immunity Melbourne VIC Australia; ^2^ National HIV and Retrovirology Laboratory National Microbiology Laboratory JC Wilt Infectious Diseases Research Centre Public Health Agency of Canada Winnipeg MB Canada; ^3^ Department of Medical Microbiology and Infectious Diseases University of Manitoba Winnipeg MB Canada; ^4^ Department of Internal Medicine University of Manitoba Winnipeg MB Canada; ^5^ Section of Nephrology Department of Internal Medicine University of Manitoba MB Canada; ^6^ Health Sciences Centre Winnipeg MB Canada; ^7^ Australian Research Council Centre for Excellence in Convergent Bio‐Nano Science and Technology University of Melbourne Melbourne VIC Australia; ^8^ Melbourne Sexual Health Centre and Department of Infectious Diseases Alfred Hospital and Central Clinical School Monash University Melbourne VIC Australia

**Keywords:** end‐stage kidney disease, glycosylation, inflammation, monocytes, tuberculosis, unconventional T cells

## Abstract

**Objectives:**

Tuberculosis comorbidity with chronic diseases including diabetes, HIV and chronic kidney disease is of rising concern. In particular, latent tuberculosis infection (LTBI) comorbidity with end‐stage kidney disease (ESKD) is associated with up to 52.5‐fold increased risk of TB reactivation to active tuberculosis infection (ATBI). The immunological mechanisms driving this significant rise in TB reactivation are poorly understood. To contribute to this understanding, we performed a comprehensive assessment of soluble and cellular immune features amongst a unique cohort of patients comorbid with ESKD and LTBI.

**Methods:**

We assessed the plasma and cellular immune profiles from patients with and without ESKD and/or LTBI (*N* = 40). We characterised antibody glycosylation, serum complement and cytokine levels. We also assessed classical and non‐classical monocytes and T cells with flow cytometry. Using a systems‐based approach, we identified key immunological features that discriminate between the different disease states.

**Results:**

Individuals with ESKD exhibited a highly inflammatory plasma profile and an activated cellular state compared with those without ESKD, including higher levels of inflammatory antibody Fc glycosylation structures and activated CX3CR1^+^ monocytes that correlate with increased inflammatory plasma cytokines. Similar elevated inflammatory signatures were also observed in ESKD^+^/LTBI^+^ compared with ESKD^−^/LTBI^+^, suggesting that ESKD induces an overwhelming inflammatory immune state. In contrast, no significant inflammatory differences were observed when comparing LTBI^+^ and LTBI^−^ individuals.

**Conclusion:**

Our study highlights the highly inflammatory state induced by ESKD. We hypothesise that this inflammatory state could contribute to the increased risk of TB reactivation in ESKD patients.

## Graphical abstract

End‐stage kidney disease (ESKD), regardless of aetiology, shares a common, highly inflammatory course promoting monocyte activation, leukocyte chemoattraction, and generalised inflammation. With substantial rates of TB reactivation in this patient group and mechanistic uncertainty, our study assesses plasma and cellular immune profiles from ESKD patients with and without latent tuberculosis infection (LTBI). We learn how ESKD^+^/LTBI^+^ co‐infection remains a highly inflammatory state compared to ESKD^−^/LTBI^+^ and find unique inflammatory signatures driven by the presence of ESKD.

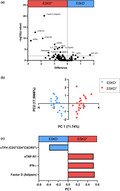

## Introduction

End‐stage kidney disease (ESKD) encompasses a range of kidney disease aetiologies, including diabetic and IgA nephropathy, which result in a common systemic state of metabolic waste accumulation, hyperuricaemia and, ultimately, dialysis‐dependent kidney failure. It is well described that chronic hyperuricaemia can impact the haemostasis of the immune system, leading to widespread dysfunction and inflammation.^1^, ^2^ The downstream effects of impaired immunity in ESKD can result in substantial comorbidities, including accelerated cardiovascular disease and susceptibility to several infectious diseases.^2^ Studies indicate that ESKD patients exhibit as much as a 52.5‐fold increase in the risk of reactivation of latent tuberculosis infection (LTBI) compared with otherwise healthy LTBI cases.^3^ The drivers of this reactivation remain unknown. Considering the rise in the prevalence of chronic kidney disease (18.4% global increase from 2005 to 2015), especially within low‐ to middle‐income countries,^4^, ^5^ where tuberculosis (TB) is also endemic, and it is critical to understand the link between ESKD and risk of TB reactivation.

The host and pathogen immune factors that lead to latency, activation and clearance of *Mycobacterium tuberculosis* (Mtb) are poorly understood. This is despite 25% of the global population living with LTBI and further increases in TB cases anticipated with COVID‐19.^6^, ^7^, ^8^ Previous studies of this unique cohort of ESKD patients, comorbid with LTBI (ESKD^+^/LTBI^+^), suggest that conventional CD4^+^ T‐cell responses to Mtb antigens are preserved in ESKD patients^9^; however, alterations in cell function of unconventional T cells may contribute to poor control of LTBI.^10^, ^11^ To date, no studies have examined the regulation of plasma cytokines, complement or antibody responses in the context of ESKD^+^/LTBI^+^.

Several lines of evidence suggest that cytokines play a critical role in the immune response to Mtb. Certain studies suggest that higher levels of IL‐12(p40), TNF‐⍺, IFN‐γ and IL‐10 have been described in patients with active TB infection (ATBI) before treatment^12^ signifying their potential role in, or as a marker of, successful Mtb infection. Osteopontin, a T helper cell 1 (Th1) cytokine secreted by macrophages, is also increased in ATBI patients.^13^ Investigation into whether ATBI‐associated inflammatory cytokines also increases in ESKD^+^/LTBI^+^ subjects would assist in characterising the immune environment in which Mtb may reactivate and replicate. Complement proteins such as C1q have been recognised as a potential biomarker for ATBI detection and may contribute to Mtb pathogenesis.^14^, ^15^ ATBI has also been associated with more inflammatory antibody glycosylation signified by agalactosylated (G0) antibodies, whereas LTBI individuals maintain antibody glycosylation states in line with those of healthy individuals.^16^, ^17^ There are no previous studies that have specifically examined Mtb‐specific antibody titres or antibody glycosylation levels in ESKD^+^/LTBI^+^ populations; hence, it is worth considering whether similar immune features observed in ATBI are also prevalent in ESKD^+^/LTBI^+^ individuals, thus signifying an environment in which Mtb replicates. Furthermore, few studies have described the frequency or activation of circulating T follicular helper (cTFH) cells in TB or ESKD, despite cTFH being a biomarker of the development of more mature serological responses to numerous infectious diseases.^18^, ^19^

Herein, we aimed to further characterise the immune defects associated with ESKD that may contribute to the elevated risk of TB reactivation. Using systems serology approaches, we assessed a large panel of plasma cytokines, chemokines, complement, antibody glycosylation and Mtb‐specific antibody profiles. We further linked these soluble plasma immune mediators to lymphocyte and monocyte subsets through the phenotypic analysis of monocytes, cTFH and unconventional T‐cell populations. Overall, we observed that patients with ESKD^+^/LTBI^+^ comorbidity exhibit a highly inflammatory plasma profile and activated cell state, which is driven by the presence of ESKD and include elevated levels of inflammatory antibody Fc glycosylation structures, complement and activated monocytes that are associated with increased plasma cytokines in comparison with ESKD^−^/LTBI^+^ individuals.

## Results

### ESKD drives distinct immune signatures regardless of LTBI disease status

To holistically examine immune signatures in ESKD^+^ patients with and without LTBI, both plasma and cellular immune responses were assessed from the following groups: ESKD alone (ESKD^+^/LTBI^−^; *n* = 10), ESKD with LTBI (ESKD^+^/LTBI^+^; *n* = 10), LTBI alone (ESKD^−^/LTBI^+^; *n* = 10) and healthy controls (ESKD^−^/LTBI^−^; *n* = 10) (Table 1). Patients with ESKD are herein referred to as ESKD^+^ (*n* = 20) and consist of both ESKD^+^/LTBI^−^ and ESKD^+^/LTBI^+^ groups, while patients without ESKD are referred to as ESKD^−^ (*n* = 20) and include both ESKD^−^/LTBI^−^ and ESKD^−^/LTBI^+^ groups. Plasma was examined for cytokines, complement levels and total IgG N‐linked glycosylation patterns. PBMCs were assessed for monocyte subsets (including classical and non‐classical) and pro‐inflammatory subsets for both traditional and unconventional T cells (including circulating T Follicular Helper (cTFH; CD4^+^CXCR5^+^ cells) and unconventional γδ lymphocytes). PBMCs were assessed by flow cytometry (see gating in Supplementary figure 1). In total, 141 immune features were assessed for each sample (Supplementary table 1). PCA (principal component analysis) of all immune features discovered divergent profiles between ESKD^+^ and ESKD^−^ patients across principal component (PC) 1 with ESKD^−^ individuals clustering negative of the *x*‐axis, while majority of ESKD^+^ were located positive (Supplementary figure 2). However, both ESKD^+^LTBI^+^ and ESKD^+^/LTBI^−^ patients overlapped together indicating similar immune profiles despite TB status.

**Table 1:** Characteristics of participants with end‐stage kidney disease and interferon gamma release assay (IGRA) status

	LTBI^−^ ESKD^−^ (*n* = 10)	LTBI^+^ ESKD^−^ (*n* = 10)	LTBI^−^ ESKD^+^ (*n* = 10)	LTBI^+^ ESKD^+^ (*n* = 10)	*P*‐value between groups
Median age (IQR), year	53.5 (39.5–59.5)	52 (39.25–53.75)	59 (49.25–69.25)	64.5 (48.75–69.75)	> 0.05
Female	6	6	7	7	> 0.05
Male	4	4	3	3	> 0.05
Canadian born	8	5	8	10	> 0.05
Non‐Canadian born	2	5	2	0	> 0.05
IGRA test^a^	0	10	0	10	‐
BCG vaccination status^b^	8	5	7	10	> 0.05
Diabetes^b^	4	1	5	6	> 0.05
Haemodialysis	0	0	10	10	‐
Cause of ESKD:	n/a	n/a			
Diabetic Nephropathy			4	6	‐
Glomerulonephritis			1	1	‐
Cystic disease			0	0	‐
Vasculitis			1	0	‐
IgA Nephropathy			1	0	‐
Cancer			0	2	‐
Other			3	1	‐

Table of patient reported data. Sex, demographics, IGRA status (laboratory confirmed), BCG vaccination status, diabetes status, treatment with haemodialysis and aetiology of ESKD (if applicable) quantitatively reported (*N* = 40). *P‐*value shows no significant differences in demographics between groups.

a Laboratory confirmed.

b Self‐reported.

### Elevated inflammatory signatures are observed in ESKD^+^ patients

**Figure 1:**
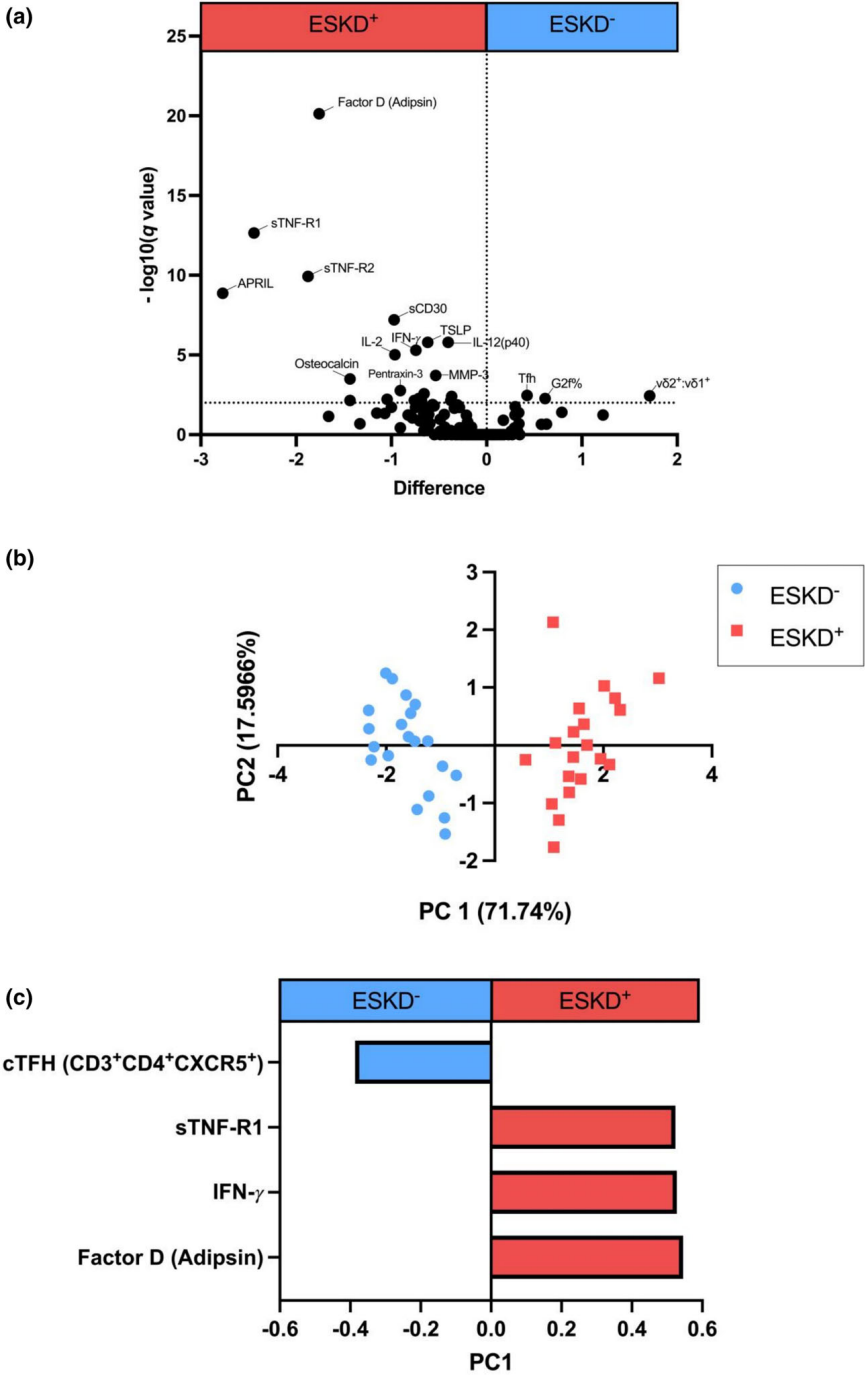
Volcano plot **(a)** utilising a multiple *t*‐test comparison between ESKD^−^ and ESKD^+^ for each measured feature assessed against the log of its *P*‐value. FDR approach using a conservative corrected method of Benjamini and Yekutieli with a desired FDR of 1%. The dotted line (*y* = 2) represents significance cut‐off specific in analysis. **(b)** Multivariant unsupervised LASSO principal component analysis (PCA) of ESKD^−^ (*n* = 20) in light blue and ESKD^+^ (*n* = 20) in light red. Separation on the scores plots indicates unsupervised separation of cohorts based on all measured features. **(c)** Loadings of principal component 1 (PC1; with 71% variance), which identify immune features capable of separating ESKD^−^ (*n* = 20) from ESKD^+^ (*n* = 20).

To distinguish the divergent immune responses between ESKD^+^ and ESKD^−^ signatures, we compared the previously mentioned 141 immune features in ESKD^−^ (*n* = 20) against ESKD^+^ (*n* = 20) (Figure 1a). 23 of 141 (16%) immune features were significantly divergent between ESKD^+^ and ESKD^−^ patients (all *P*‐values < 0.0003, corrected for multiple comparisons, Supplementary table 1). Features upregulated in ESKD^+^ plasma included the following factors and cytokines (Figure 1a; Supplementary table 1): complement Factor D (Adipsin) (median 7 ng mL^−1^ in ESKD^−^ cf. median 45 ng mL^−1^ in ESKD^+^), soluble TNF‐receptor 1 (sTNF‐R1; 2341 pg mL^−1^ in ESKD^−^ cf. 29363 pg mL^−1^ in ESKD^+^), soluble TNF Receptor 2 (sTNF‐R2; 1345 pg mL^−1^ in ESKD^−^ cf. 8380 pg mL^−1^ in ESKD^+^), APRIL (122852 pg mL^−1^ in ESKD^−^ cf. 2050367 pg mL^−1^ in ESKD^+^), sCD30 (909 pg mL^−1^ in ESKD^−^ cf. 2169 pg mL^−1^ in ESKD^+^), TSLP (98 pg mL^−1^ in ESKD^−^ cf. 186 pg mL‐1 in ESKD^+^), IL‐12(p40) (255 pg mL^−1^ in ESKD^−^ cf. 349 pg mL^−1^ in ESKD^+^), IFN‐γ (151 pg mL^−1^ in ESKD^−^ cf. 269 pg mL^−1^ in ESKD^+^), IL‐2 (10 pg mL^−1^ in ESKD^−^ cf. 24 pg mL^−1^ in ESKD^+^), MMP‐3 (5812 pg mL^−1^ in ESKD^−^ cf. 11518 pg mL^−1^ in ESKD^+^), Osteocalcin (6531 pg mL^−1^ in ESKD^−^ cf. 28879 pg mL^−1^ in ESKD^+^), Pentraxin‐3 (2235 pg mL^−1^ in ESKD^−^ cf. 5397 pg mL^−1^ in ESKD^+^) and IL‐29/IFN‐n1 (69 pg mL^−1^ in ESKD^−^ cf. 140 pg mL^−1^ in ESKD^+^). Of interest, many of these inflammatory cytokines including sTNF‐R1, sTNF‐R2, IFN‐γ and MMP‐3 have also positively correlated with clinical severity of ATBI^20^, ^21^, ^22^ and IFN‐γ has been associated with Mtb infection activity as measured through radiologically determined pulmonary infiltrates and destruction.^23^ TNF‐R1 has also been identified in previous studies as a marker of rapid ESKD progression.^24^ Significantly elevated levels of agalactosylated (G0) N‐linked Fc antibody glycans were observed in ESKD^+^ patients in comparison with ESKD^−^ (median 3 in ESKD^−^ cf. 6.5 in ESKD^+^; *P* = 0.0016). Of interest, elevated agalactosylated antibodies have also been associated with ATBI in comparison with LTBI.^16^

CD14^+^CD16^−^ monocytes expressing CX3CR1 were higher in the ESKD^+^ group (median fluorescent intensity (MFI); 2605 in ESKD^−^ cf. 3523 in ESKD^+^), which have previously been used to measure innate immune inflammatory phenotypes in ESKD^+^ populations.^11^ Effector memory (CD27^−^) CD4^+^ T cells were also elevated in the ESKD^+^ cohort (median; 5% of bulk CD4^+^ T cells in ESKD^−^ cf. 14% in ESKD^+^) (Supplementary table 1). In comparison, G2f% galactosylation (median 13 in ESKD^−^ cf. 6 in ESKD^+^; *P* = 0.00043) of bulk IgG antibodies was upregulated in the ESKD^−^ group, which has previously been associated with LTBI.^16^ Total frequencies of cTFH cells (median 29% of memory CD4^+^ in ESKD^−^ cf. 19% in ESKD^+^) and Vδ2:Vδ1 T cell ratio were also significantly higher in the ESKD^−^ group (median ratio of 4.6 in ESKD^−^ cf. 0.4 in ESKD^+^) (Figure 1a).

### **Four minimal immune features distinguish ESKD^+^ from ESKD** ^−^ **patients**

To further characterise the impact of ESKD on the immune state, we applied multivariate computational analysis, including feature reduction to identify the minimal immune profile that distinguished ESKD^−^ from ESKD^+^ (Figure 1b). Strikingly, only 4 key immune features were selected that differentiated ESKD^−^ from ESKD^+^ individuals (Figure 1c). These features included elevated TNF‐R1, IFN‐γ and Factor D (Adipsin) within ESKD^+^ individuals and elevated cTFH (CD3^+^CD4^+^CXCR5^+^) frequencies in ESKD^−^ (median 6.4% in ESKD^+^ cf. 12.8% in ESKD^−^; Supplementary figure 3).

### Inflammatory antibody glycosylation and complement profiles distinguish ESKD^+^LTBI^+^ and ESKD^−^LTBI^+^ individuals

**Figure 2:**
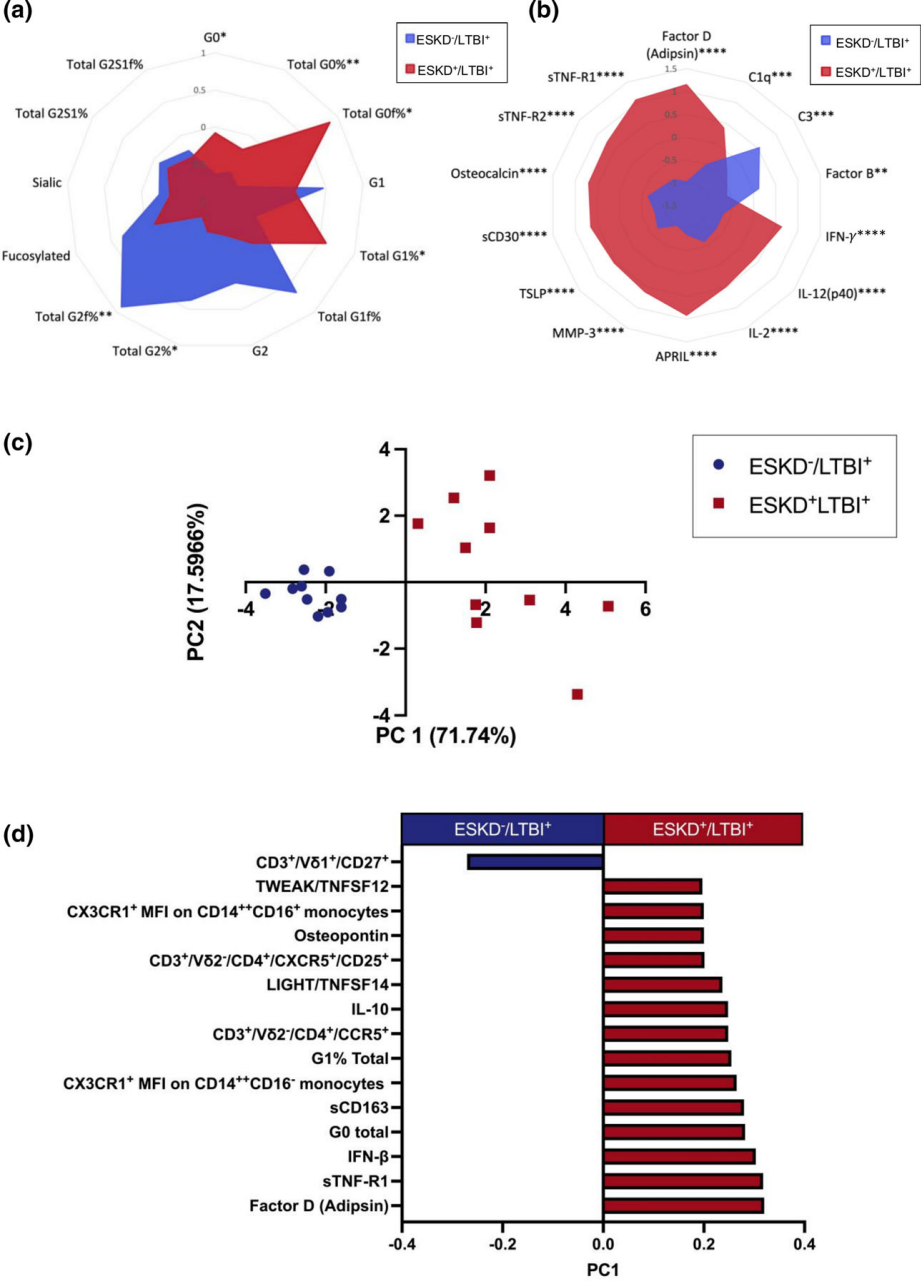
Radar plots of **(a)** glycosylation patterns of purified IgG from LTBI^+^ (*n* = 10) and ESKD^+^/LTBI^+^ (*n* = 10) serum (glycans measured as total area under the curve; the sum of total glycan area peaks with % make‐up of each glycan measured as total %) and **(b)** complement serum levels in LTBI^+^ (*n* = 10) and ESKD^+^/LTBI^+^ (*n* = 10) patients (MFI). All data *Z*‐scores are normalised. Differences between groups were analysed with unpaired two‐tailed *t*‐tests, with *P < 0.05, **P < 0.01, ***P < 0.001, ****P < 0.0001. **(c)** Unsupervised LASSO PCA differentiating ESKD^−^/LTBI^+^ (*n* = 10) and ESKD^+^/LTBI^+^ (*n* = 10) with the immune features and their loadings associated with this separation **(d)**.

Previous studies have identified elevated inflammatory N‐linked antibody glycosylation and complement levels as biomarkers of ATBI in comparison with LTBI.^16^, ^25^ Therefore, we examined ESKD^−^/LTBI^+^ (*n* = 10) and ESKD^+^/LTBI^+^ (*n* = 10) patients for differences in N‐linked glycans (Figure 2a) and complement (Figure 2b). Total agalactosylated IgG (Total G0%) was higher in ESKD^+^/LTBI^+^ patients (*P* = 0.015), consistent with elevated G0 (*P* = 0.006) and G0f structures (*P* = 0.011) (Figure 2a), whereas di‐galactosylated IgG (G2 and G2f) was increased in ESKD^−^/LTBI^+^ individuals (G2f% *P* = 0.0019; G2% *P* = 0.012; Figure 2a). ESKD^+^/LTBI^+^ patients also exhibited significantly higher complement Factor D (Adipsin) (*P* < 0.0001) and C1q (*P* = 0.009) (Figure 2b), whereas ESKD^−^ individuals had significantly higher C3 (*P* = 0.012) and Factor B (*P* = 0.0078) (Figure 2b).

Given that previous studies have observed differential antibody responses in TB disease states,^16^ we next assessed Mtb‐specific antibodies in all LTBI patients via a customised Mtb‐specific multiplex assay including 16 TB antigens and influenza HA as a positive control. We measured antigen‐specific antibody isotype (IgG, IgA) and subclass (IgG1, IgG2, IgG3, IgG4; IgA1, IgA2) levels, such that a composite antibody database of 119 antigen‐specific antibody features was compiled (17 antigens x 7 detectors). Surprisingly, there were near‐undetectable IgG responses to Mtb‐specific antigens despite detectable levels of influenza‐specific IgG (Supplementary figure 4). This suggests an ability for antigen‐specific IgG (i.e. influenza‐specific IgG) to be generated by ESKD^+^ patients; however, the antigenic stimulation present with LTBI in this cohort may be too low to induce robust Mtb antibody responses, a finding that is consistent with previous studies demonstrating a correlation between Mtb antibody titres and the level of Mtb bacterial burden.^26^, ^27^

To further examine the influence of ESKD upon immune responses in LTBI patients, we compared all immune features (a total of 260 features: 141 afore‐mentioned immune features along with 119 antibody responses) between LTBI^+^ (ESKD^+^/LTBI^+^ with ESKD^−^/LTBI^+^ *n* = 20) and LTBI^−^ (ESKD^−^/LTBI^−^ healthy controls with ESKD^+^/LTBI^−^; *n* = 20). No significant differences were identified between groups, after taking into account multiple comparisons (data not shown). We next applied feature selection to determine the minimal immune signatures that distinguish ESKD^+^/LTBI^+^ and ESKD^−^/LTBI^+^ patients (Figure 2c). In addition to 3 of the features identified in the previous analysis shown in Figure 1c, another 12 immune features were selected (IFN‐γ was not identified; Figure 2d). Interestingly, despite prior observations of functional changes in Vδ2 T cells among the ESKD^+^LTBI cohort,^10^ the systems analysis identified the frequency of CD27^+^ Vδ1 T cells as the only cellular immune feature elevated in the ESKD^−^/LTBI^+^ group, whereas CX3CR1^+^CD14^++^CD16^+^ and CD14^++^CD16^−^ monocytes, CD3^+^/Vδ2^−^/CD4^+^/CCR5^+^ and CD3^+^Vδ2^−^/CD4^+^CXCR5^+^/CD25^+^ cells were elevated in ESKD^+^/LTBI^+^ patients. In addition, inflammatory cytokines (TWEAK/TNFSF12, Osteopontin, IFN‐beta, LIGHT/TNFSF14, IL‐10 and sCD163), complement protein Factor D (Adipsin) and antibody glycosylation patterns G0 and G1 (Figure 2d) were also elevated in ESKD^+^/LTBI^+^ individuals. Overall, this confirmed an elevated inflammatory signature in ESKD^+^/LTBI^+^ patients.

### CX3CR1^+^ monocytes in ESKD^+^ individuals correlate with elevated inflammatory plasma cytokines

**Figure 3:**
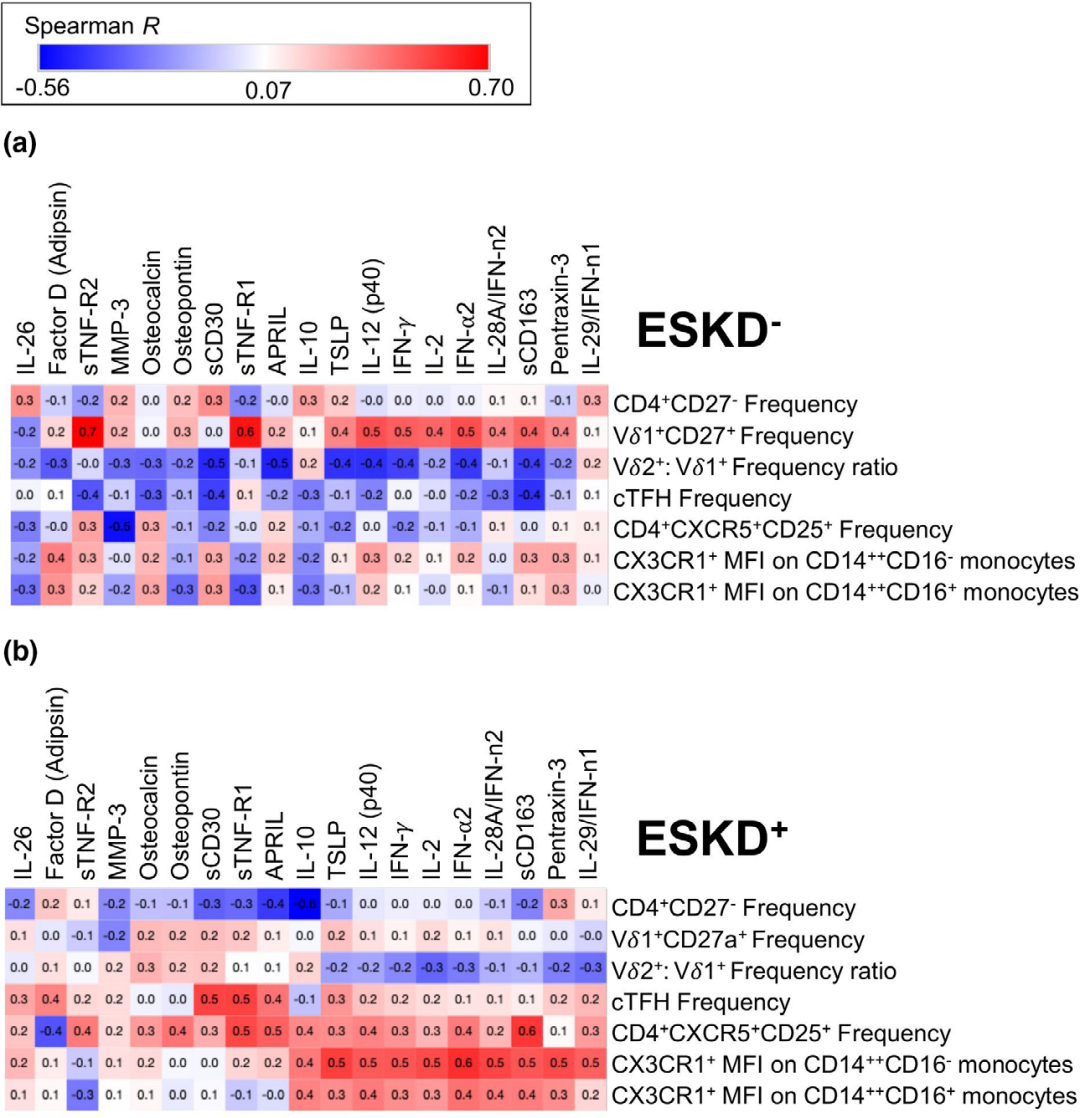
Correlation plots of non‐parametric Spearman’s correlation. *y*‐axis CD4^+^, Vδ1^+^ and Vδ2^+^/Vδ1^+^ ratios, cTFH cells and monocytes. *x*‐axis cytokines of significance are highlighted in Figures 1 and 2. **(a)** (ESKD^−^ *R*‐values; min –0.56 to max 0.70); **(b)** (ESKD^+^ *R*‐values); *R* ≥ 0.446 are significant *P* < 0.05.

Many of the cytokines highlighted thus far, are produced by and/or are chemoattractants for cells critical to the Mtb response including monocytes and CD4^+^ T cells. Hence, we correlated the previous feature selected cytokines with cell phenotypes (Figure 3a, b). In ESKD^+^ patients (Figure 3b), a cluster of pro‐inflammatory cytokines (TSLP, IL‐12(p40), IFN‐γ, IL‐2, IFN‐⍺2, IL‐28A/IFN‐n2, sCD163, Pentraxin‐3 and IL‐29/IFN‐n1) correlated positively with expression of CX3CR1^+^ on CD14^++^CD16^−^ and CD14^++^CD16^+^ monocytes (which were elevated in ESKD^+^; Supplementary figure 5a–c) as well as CD4^+^CXCR5^+^CD25^+^ T cells, while no significant correlations were observed in the ESKD^−^ cohort. cTFH cell frequency also correlated significantly with sCD30 and sTNF‐R1 within ESKD^+^ patients. In contrast, Vδ1^+^CD27^+^ frequency was positively correlated with the cluster of cytokines listed above within ESKD^−^ patients, but interestingly did not correlate within the ESKD^+^ cohort. Overall, these results suggest that different monocyte and T‐cell profiles contribute to the elevated inflammatory signatures observed in ESKD^+^ patients.

## Discussion

Considering the prevalence of Mtb and the rise in chronic kidney disease across the globe, understanding the influence of this common disease upon Mtb^28^ is of great public health importance. In this study, we found ESKD^+^ individuals demonstrated an elevated pro‐inflammatory signature in comparison with ESKD^−^ individuals, including a panel of inflammatory plasma signatures comprising cytokines, complement and Fc glycosylation along with monocyte and T‐cell subsets. Furthermore, within the ESKD^+^ cohort, pro‐inflammatory cytokine levels were found to correlate with activated monocytes expressing CX3CR1, whereas no significant correlations to activated monocytes were observed within the ESKD^−^ cohort. Similar elevated inflammatory signatures were also observed in ESKD^+^/LTBI^+^ versus ESKD^−^/LTBI^+^, strongly suggesting that the ESKD^+^/LTBI^+^ inflammatory response was driven by ESKD^+^.

The uremic state of chronic kidney disease and ESKD can impact monocyte function, where their dysregulated inflammatory activity has been attributed to vascular damage.^29^ CX3CR1 expression is recognised as a mediator of disease severity in kidney disease, with immunotherapeutic inhibition of CX3CR1 shown effective against glomerulonephritis.^30^, ^31^, ^32^ Elevated levels of CX3CR1 are also observed in HIV‐Mtb co‐infection, another disease where Mtb reactivation is of great burden.^33^ Similarly, CD14^++^CD16^−^ monocytes, which were also elevated in both our ESKD^+^ and ESKD^+^/LTB^+^ cohorts, have previously been identified as predictors of Mtb‐associated immune reconstitution inflammatory syndrome.^34^

In this present study, we observed the same elevated cytokines including sCD163, Osteopontin and Pentraxin‐3 in both the ESKD^+^ cohort and the ESKD^+^/LTB^+^ subgroups. These cytokines can be secreted or shed by inflammatory activated macrophages and are involved in the activation of monocytes or inflammatory cells nearby.^35^, ^36^, ^37^, ^38^ Notably, Osteopontin levels are greater in ATBI than in LTBI or healthy individuals.^13^ Given the role that monocytes play in TB infection and dissemination,^39^, ^40^ and the high Mtb reactivation rates in ESKD^+^ patients, it is worth considering how this inflammatory state influences the pulmonary microenvironment and subsequent emergence of fulminant TB. Collectively, our study suggests a picture of overwhelming pro‐inflammatory responses in patients with ESKD, which supports the recent postulation that the severity of TB in patients comorbid with diabetes is due to excessive inflammation and monocyte activation.^41^ Future studies examining monocyte function including cytokine secretion (e.g. stimulated with Mtb or BCG) from individuals with different disease states would be of great interest to confirm this hypothesis.

Antibody glycosylation levels of G0, the agalactosylated form of N‐glycans was higher in ESKD^+^ patients in this study. G0 has been associated with inflammatory autoimmune conditions such as rheumatoid arthritis and is also elevated in people with diabetes mellitus.^42^, ^43^, ^44^ Previous studies have found that G0 levels are associated with ATBI.^16^ Given that ESKD patients also have elevated G0 levels, we hypothesise that a similar mechanism of inflammation is induced in both ESKD and ATBI.^16^

Changes in bulk antibody glycosylation profiles, together with reduced cTFH frequencies in ESKD, may also reflect underlying perturbations of B‐cell responses in secondary lymphoid organs. To date, we are unaware of other studies assessing cTFH in the context of ESKD^+^ or ESKD^+^/LTBI^+^. In addition to cTFH frequency, differences in the memory phenotype of Vδ1 T cells were highlighted as a feature differentiating ESKD^−^ and ESKD^+^ individuals with LTBI. Differentiation of Vδ1 T cells towards a CD27^−^ phenotype has previously been reported in CMV infection and is associated with clonal expansion and upregulation of cytotoxic mediators such as perforin and granzyme.^45^ Within the ESKD^−^ cohort, CD27^+^ Vδ1^+^ cells were the only cell type to positively correlate with the same set of inflammatory cytokines that correlate with monocytes, and cTFH frequency/activation in the ESKD^+^ group. Interestingly, other inflammatory diseases associated with microbial translocation (including HIV) similarly result in the expansion of CD27^−^ Vδ1 T‐cell populations.^46^ This begs the question as to whether ESKD‐associated inflammation drives Vδ1 T cells towards a differentiated, CD27^−^ phenotype.

Caveats to this study include small sample size, the inability to collect samples from patients with ESKD where LTBI reactivated, due to rapid mortality following reactivation. As this was a cross‐sectional study, we cannot determine whether LTBI treatment or improvements in patients' kidney function impact the levels of these inflammatory markers. Most patients within this cohort were on several different medications to treat ESKD, which can affect their serological and cellular immune responses, and potentially influence LTBI reactivation, such as corticosteroids. Due to the size and heterogeneity of the cohort, these medications were not controlled; however, large cohort studies where the influence of medications upon inflammation and reactivation is monitored should be investigated in the future studies.

This study brings together multiple concepts relating to co‐infection, inflammation and plasma markers in the context of ESKD and LTBI. ESKD, regardless of the aetiology, shares a common, highly inflammatory course that promotes monocyte activation, leukocyte chemoattraction and generalised inflammation. Given the substantial rates of TB reactivation in this patient group and the mechanistic uncertainty, our study furthers the understanding that ESKD^+^/LTBI^+^ co‐infection remains a highly inflammatory state compared with ESKD^−^/LTBI^+^, with the highly inflammatory state driven by the presence of ESKD. Identifying the mechanisms of LTBI reactivation in these patients may lead to pharmacological agents that can block the action of these specific cytokines or complement proteins, preventing the facilitation of reactivation. Further mechanistic research into this area must be conducted and larger patient cohorts enlisted.

## Methods

### Study participants

This cohort of study participants with ESKD, LTBI and healthy controls has been previously described.^9^, ^10^, ^11^ Individuals living with ESKD and undergoing haemodialysis were recruited as part of the Renal Program at the Health Sciences Centre in Manitoba, Canada. ESKD^−^ control PBMCs and plasma were selected from a TB immunology biobank in Manitoba. ESKD^−^ controls were demographically matched in location and diabetic status where possible. All participants were administered the QuantiFERON‐TB Gold In‐Tube™ Test (Qiagen, Hilden, Germany) and were HIV, HBV and HCV negative at the time of participation. All participants provided informed consent. The study was approved by the Research Ethics Board at the University of Manitoba.

### Peripheral blood collection and processing

Peripheral blood samples were collected for plasma and PBMC collection. Plasma was stored at –80°C in aliquots for antibody, cytokine and complement determination. PBMCs were isolated via Ficoll (Bio‐Strategy Lab, Melbourne, Australia) gradient separation and cryopreserved prior to stimulation and culture.

### Antibody purification

IgG was purified from 100 µL of IgA‐depleted plasma using Melon Gel IgG Purification method (Thermo Fisher, Massachusetts, USA). Purified IgG concentrations were confirmed via ELISA.

### Profiling of IgG N‐linked glycans

Glycan analysis was performed on purified IgG using the LabChip GXII Touch protein characterisation system (PerkinElmer, Massachusetts, USA). To determine glycan glycosylation samples were processed using a HT Protein Express Reagent Kit (PerkinElmer, Massachusetts, USA) according to the manufacturer's instructions (protocol CLS140171). For glycan profiling, samples were processed using the ProfilerPro Glycan Profiling Assay Kit (PerkinElmer, Massachusetts, USA). Briefly, samples were reduced and digested with PNGase F to release N‐glycans, and the N‐glycans fluorescently labelled. Glycans were identified using a panel of commercially available N‐glycan standards (QA‐Bio, California, USA), and the proportion of each glycan was profiled with the LabChip GX Reviewer software (PerkinElmer, Massachusetts, USA).

### Systems serology

#### Multiplex bead‐based human inflammation assay

The plasma as described above from the study samples (*N* = 40) was assessed using the Bio‐Plex Pro™ Human Inflammation Panel 1, 37‐Plex #171AL001 M (Bio‐Rad, California, USA) on a Luminex FlexMap 3D machine (Luminex, Texas, USA), following the manufacturer’s recommendations at a 1:2 plasma dilution. The following 37 analytes were assessed: APRIL/TNFSF13, BAFF/TNFSF13B, sCD30/TNFRSF8, sCD163, Chitinase‐3‐like 1, gp130/sIL‐6Rβ, IFN‐α2, IFN‐β, IFN‐γ, IL‐2, sIL‐6Rα, IL‐8, IL‐10, IL‐11, IL‐12 (p40), IL‐12 (p70), IL‐19, IL‐20, IL‐22, IL‐26, IL‐27 (p28), IL‐28A/IFN‐λ2, IL‐29/IFN‐λ1, IL‐32, IL‐34, IL‐35, LIGHT/TNFSF14, MMP‐1, MMP‐2, MMP‐3, Osteocalcin, Osteopontin, Pentraxin‐3, sTNF‐R1, sTNF‐R2, TSLP, TWEAK/TNFSF12. Thirty‐six of the 37 analytes assayed were included in analyses as IL‐20 showed undetectable levels in all plasma samples.

#### Multiplex bead‐based complement assay

Plasma study samples were also assessed for complement using the Merck Millipore Milliplex Human Complement Panel 1 (HCMP1MAG‐19K) and Human Complement Panel 2 (HCMP2MAG19K) (Merck Millipore, Massachusetts, USA), following the manufacturer’s recommendations on a Luminex FlexMap 3D. The plasma samples were prepared at a dilution of 1:200 as recommended for Panel 1 and 1:40 000 as recommended by Panel 2. The following analytes were assessed in Panel 1: C2, C4b, C5, C9, Factor D (Adipsin), Mannose Binding Lectin (MBL) and Factor I. The following analytes were assessed in Panel 2: C1q, C3, C3b/iC3b, C4, Factor B, Factor H and Properdin.

#### Tuberculosis and influenza‐specific antibody multiplex assay

A customised TB multiplex assay was designed to assess for Ab specific for Ag85B, MPT64, TX114 proteins, TB peptidoglycan, Ag85 complex and ESAT‐6 (BEI resources, Manassas, USA). ATBI‐positive serum from a different cohort was used as a control to verify Mtb antigen binding. H3 influenza haemagglutinin (Sino Biological A/Switzerland/9715293/2013, HA; Sino Biological, Beijing, China) was also included in the array as a positive antigen control. Briefly, magnetic carboxylated beads (Bio‐Rad, California, USA) were covalently coupled to Mtb and Flu antigens by carbodiimide reaction as previously described.^47^ The isotypes and subclasses (IgG, IgA1, IgA2, IgG1‐4) of antigen‐specific Abs were assessed as previously described.^48^

### Flow cytometry

PBMCs were thawed and stained as previously described.^10^, ^11^ Briefly, cryopreserved PBMC were thawed, stained with live dead blue and incubated with a cocktail of surface antibodies for 30 min at 4°C. Cells were then washed, fixed in BD Cytofix/Cytoperm (BD, New Jersey, USA) and acquired on a BD LSR Fortessa using BD FACS Diva (BD, New Jersey). Data were analysed in FlowJo v10 (FlowJo, Oregon, USA). Gates were set according to fluorescent minus one controls. Surface antibody cocktails (BioLegend, California, USA): CX3CR1 FITC (2A9‐1), CD14 PerCP‐Cy5.5 (MOP9), CD16 AlexaFluor700 (3G8), HLA‐DR APC‐Fire750 (L243), CCR2 BV421 (48607), CD3 BV510 (SK7), CD4 BV605 (RPA‐T4), CD8 BV650 (RPA‐T8), CD11b BV785 (ICRF44), Vδ2 PE (B6), CD20 Pe‐Dazzle594 (2H7), CD56 BUV395 (NCAM16.2), CXCR5 BB515 (RF8B2), CCR7 Alexa647 (G043H7), CD25 APC‐R700 (2A3), CD69 APC‐Fire750 (FN50), PD‐1 BV421 (EH12.2H7), CCR6 BV785 (G034E3), CXCR3 PE‐Dazzle594 (G02H57) and CD45RA PeCy7 (HI100).

### Statistical analysis

#### Data normalisation

For all multivariate analysis, influenza positive controls were removed. Right shifting was performed on each immune feature if any negative values were observed by adding the minimum value of the feature back to all samples. For PCA, to transform the features to have a normal distribution, data were log‐transformed following the equation *y* = log_10_ (*x* + 1), where *x* is the right‐shifted data. Data were further normalised by mean centring and variance scaling.

#### Feature selection

Key immune features (signatures) that contributed to differences between cohorts were identified using the least absolute shrinkage and selection operator (LASSO) penalised regression feature selection method in MATLAB (MathWorks, Massachusetts, USA) using the statistics and machine learning tool box.^47^ Cross‐validation was performed iteratively (repeated 10 000 times, 10‐fold cross‐validation) to find the optimal regularised parameters.

#### PCA

Principal component analysis (PCA) was performed in MATLAB using the statistic and machine learning toolbox, in order to visualise the variance of all measured features for each sample. Each measure immune feature is assigned a loading, with the linear combinations of these loadings forming the observed principal component (PC). Each sample is scored using their individual measured immune responses and plotted. Separation on the scores plots indicates unsupervised separation of cohorts based on all measured features.

### Software

Univariate analyses were performed using GraphPad Prism 9 software (GraphPad, California) with normalisation of data pre‐analysis with MATLAB scripts. Univariate analyses were unpaired and did not assume normal distributions. Data normalisation, feature selection and PCA were completed using MATLAB with statistics and machine learning toolbox (MathWorks, Massachusetts, USA). PCA scores and loading plots were graphed in Prism.

## Conflicts of interest

The authors declare no conflict of interest.

## Author contribution

**Milla Rose McLean:** Data curation; Formal analysis; Investigation; Methodology; Project administration; Software; Validation; Visualization; Writing‐original draft; Writing‐review & editing. **Kathleen M Wragg:** Data curation; Formal analysis; Methodology; Writing‐review & editing. **Ester Lopez:** Data curation; Methodology; Supervision; Writing‐review & editing. **Sandra A Kiazyk:** Project administration; Resources; Writing‐review & editing. **Terry B Ball:** Funding acquisition; Project administration; Resources; Writing‐review & editing. **Joe Bueti:** Project administration; Resources; Writing‐review & editing. **Stephen Kent:** Funding acquisition; Project administration; Resources; Writing‐review & editing. **Jennifer Juno:** Conceptualization; Data curation; Formal analysis; Funding acquisition; Investigation; Methodology; Project administration; Resources; Software; Supervision; Validation; Visualization; Writing‐original draft; Writing‐review & editing. **Amy Chung:** Conceptualization; Data curation; Formal analysis; Funding acquisition; Investigation; Methodology; Project administration; Resources; Software; Supervision; Validation; Visualization; Writing‐original draft; Writing‐review & editing.

## Supporting information

Supplementary figures 1–5
Supplementary table 1
