# Supplementary material for: Serological and cellular inflammatory signatures in end‐stage kidney disease and latent tuberculosis
Source: Clin Transl Immunology. 2021 Nov 5;10(11):e1355. doi: 10.1002/cti2.1355 (PMC8569694; doi:10.1002/cti2.1355)
Supplement: Supplementary file 1 — Supplementary figures 1–5 Supplementary table 1 [file CTI2-10-e1355-s001.pdf]

Supplementary figure 1

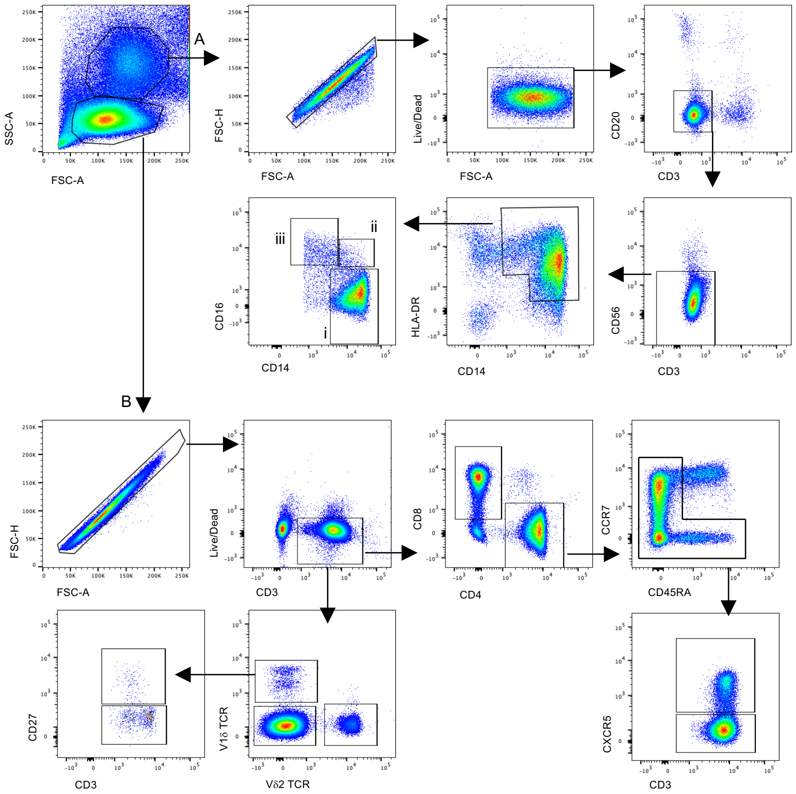

Supplementary figure 1. Flow cytometry gating strategy. Monocytes (A) and lymphocytes (B) were identified by forward and side scatter (FSC vs SSC). After exclusion of doublets and dead cells, monocytes were identified as CD3-CD20-CD56^{-} HLA^{-}DR^{+}CD14^{+} cells, and further categorized as (i) CD14^{++}CD16^{-}, (ii) CD14^{++}CD16^{+} or (iii) CD14^{+}CD16^{++}. (B) After exclusion of doublets and dead cells, gamma delta T cells were defined as CD3^{+}Vδ1^{+} TCR^{+} or Vδ2^{+} TCR^{+}, with phenotyping for CD27 expression. Conventional T cells were identified as CD8+ or CD4^{+}. CD4^{+} T cells were categorized as naïve (CCR7^{+}CD45RA^{+}) or non-naïve, which included cTFH cells (CXCR5^{+}).

Supplementary table 1

Supplementary table 1. 23 out of 141 Discoveries from volcano plot utilizing a False-Discovery Rate approach. *P*-value was corrected for multiple comparisons using the Corrected FRD Method. Desired FDR (Q) 1.00%.

	P-value	Mean of ESKD- (log transformed)	Mean of ESKD+ (log transformed)	Difference	SE of difference	*t*-ratio	df	*q*-value
Factor D (Adipsin)	<0.000001	1.956	3.715	-1.759	0.07726	22.77	38.00	<0.000001
sTNF-R1	<0.000001	7.648	10.09	-2.442	0.1823	13.39	38.00	<0.000001
sTNF-R2	<0.000001	7.193	9.068	-1.876	0.1746	10.74	38.00	<0.000001
APRIL	<0.000001	11.45	14.22	-2.770	0.2841	9.748	38.00	<0.000001
sCD30	<0.000001	6.784	7.755	-0.9715	0.1164	8.347	38.00	<0.000001
TSLP	<0.000001	4.538	5.156	-0.6181	0.08561	7.220	38.00	0.000002
IL-12(p40)	<0.000001	5.517	5.921	-0.4044	0.05641	7.169	38.00	0.000002
IFN-γ	<0.000001	4.841	5.583	-0.7418	0.1097	6.760	38.00	0.000005
IL-2	<0.000001	2.198	3.162	-0.9636	0.1479	6.516	38.00	0.000010
MMP-3	0.000002	8.810	9.345	-0.5353	0.09664	5.539	38.00	0.000190
Osteocalcin	0.000004	8.669	10.10	-1.435	0.2683	5.349	38.00	0.000315
Pentraxin-3	0.000026	7.625	8.531	-0.9059	0.1896	4.779	38.00	0.001719
IL-29/IFN-n1	0.000045	4.295	4.953	-0.6580	0.1428	4.608	38.00	0.002683
cTFH Frequency	0.000062	3.336	2.911	0.4247	0.09428	4.505	38.00	0.003425
$\gamma\delta$1 T cell frequency	0.000069	1.383	-0.3294	1.712	0.3834	4.465	38.00	0.003611
CX3CR1 MFI on $CD14^{++}CD16^{-}$ monocytes	0.000081	7.860	8.227	-0.3677	0.08330	4.414	38.00	0.003957
G2f %Area	0.000122	2.426	1.812	0.6137	0.1434	4.279	38.00	0.005431
G0 total	0.000125	1.227	1.939	-0.7119	0.1667	4.271	38.00	0.005431
$CD4^{+}$ T cells, Frequency of $CD27^{-}$ T_{EM}	0.000147	1.900	2.945	-1.045	0.2476	4.218	38.00	0.006028
Osteopontin	0.000186	12.36	13.80	-1.434	0.3463	4.141	38.00	0.007236
IL-26	0.000196	5.746	6.504	-0.7581	0.1839	4.122	38.00	0.007239
IFN-a2	0.000204	4.039	4.416	-0.3765	0.09163	4.109	38.00	0.007239
IL-28A/IFN-n2	0.000228	2.664	3.347	-0.6826	0.1677	4.071	38.00	0.007744

Supplementary figure 2

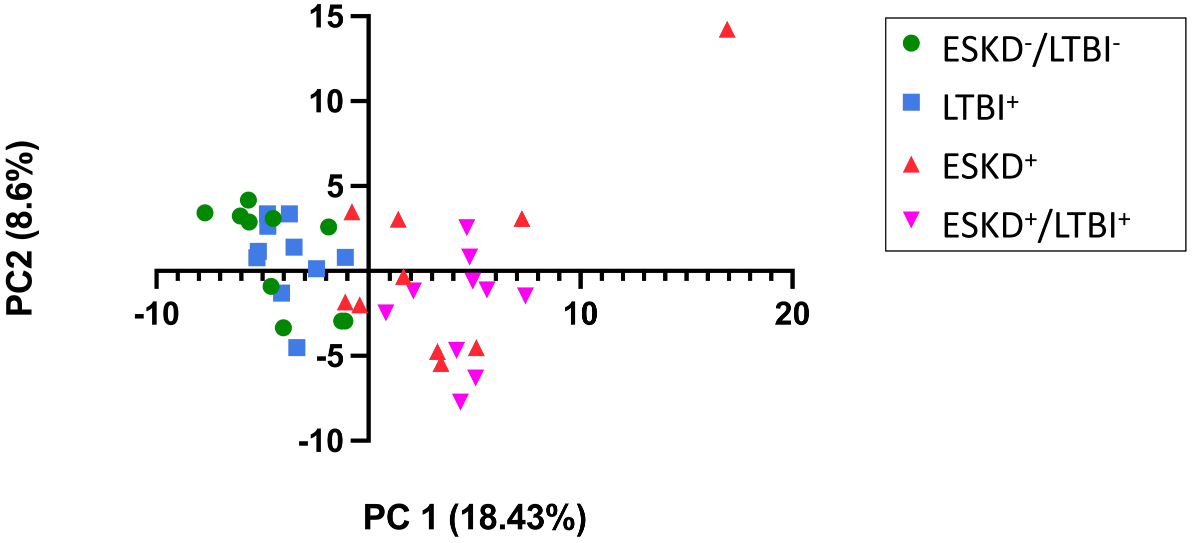

Supplementary figure 2. Multivariant unsupervised LASSO Principal Component (PC) Analysis (PCA) of ESKD$^-$/LTBI$^-$ (*n* = 10; green)), LTBI$^+$ (*n* = 10; blue), ESKD$^+$ (*n* = 10; red) and ESKD$^+$/LTBI$^+$ groups (*n* = 10; pink)

Supplementary figure 3

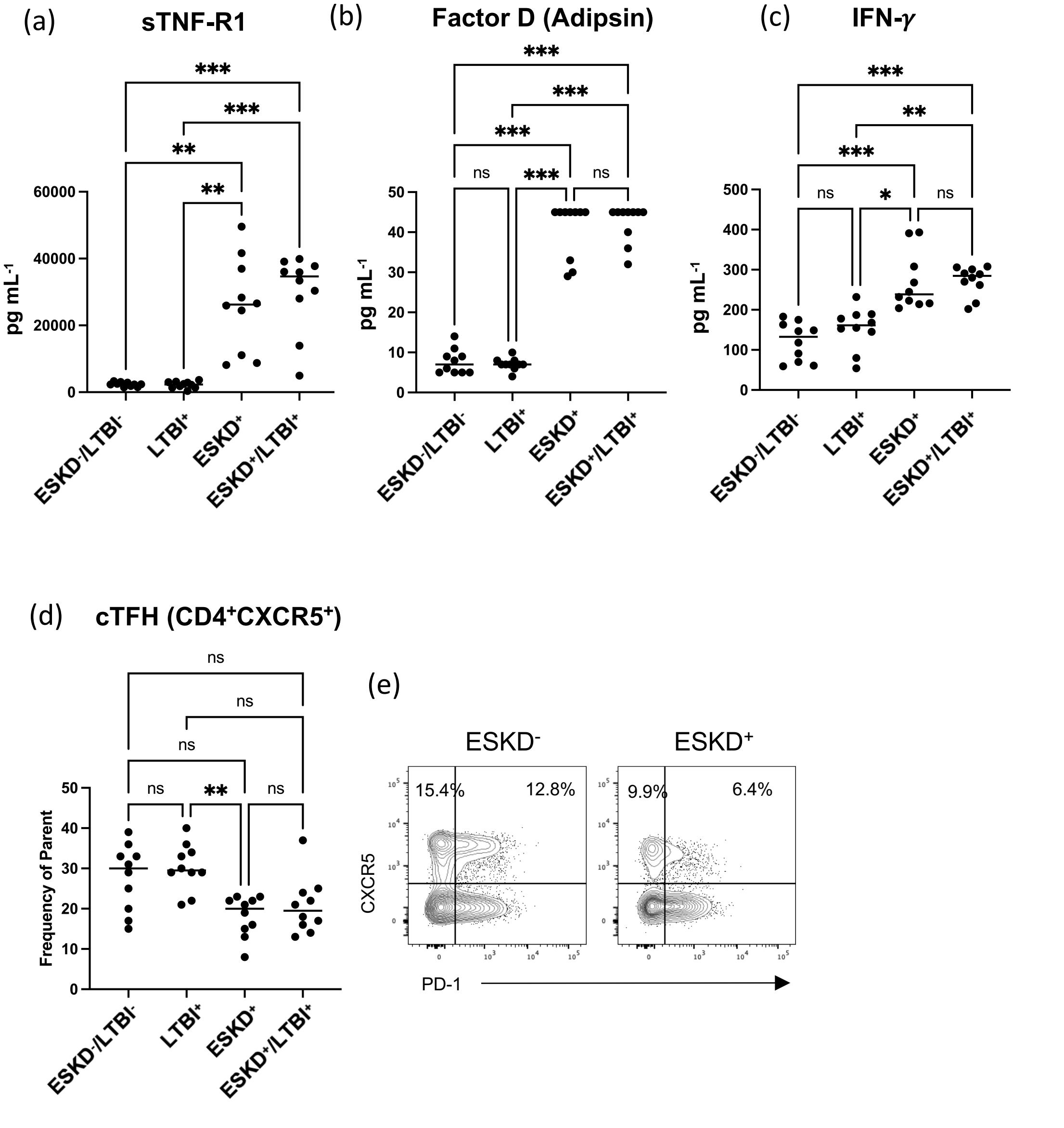

Supplementary figure 3. Individual plots of 4 key features selected by LASSO PCA analysis (sTNF-R1 **(a)**, Factor D (Adipsin) **(b)**, IFN-γ **(c)** and cTFH (CD4$^+$CXCR5$^+$) Frequency **(d)**) comparing pg mL^{-1} in each respective group (ESKD$^-$/LTBI$^-$ (n = 10), LTBI$^+$ (n = 10), ESKD$^+$ (n = 10), ESKD$^+$/LTBI$^+$ (n = 10); total n = 40). Median of each group shown as line bar in plots. **(e)** Separation of PD1$^+$ (x-axis) and CXCR5$^+$ (y-axis) CD4$^+$ TFH cells via cell-plots.

Supplementary figure 4

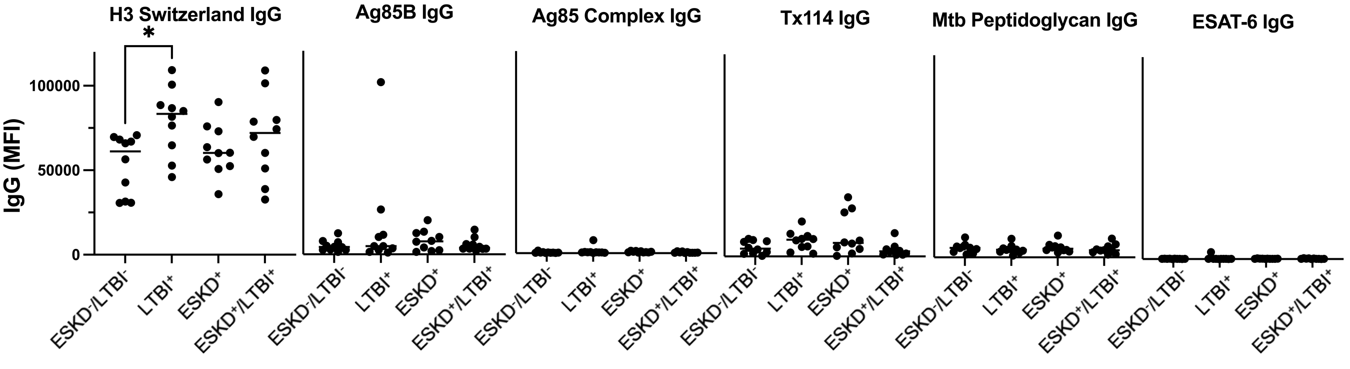

Supplementary figure 4. Multiplex assay univariant analysis reporting mean fluorescence intensity (MFI; linear scale) of Influenza and TB-specific IgG in ESKD$^-$/LTBI$^-$ *(n* = 10), LTBI$^+$ (*n* = 10), ESKD$^+$ (*n* = 10), ESKD$^+$/LTBI$^+$ (*n* =10). Line bar indicates median. 5 antigens chosen to represent total of 16 assessed due to these five antigens representing internal and external TB antigens. 11 other antigens had similar MFIs and were not significant.

Supplementary figure 5

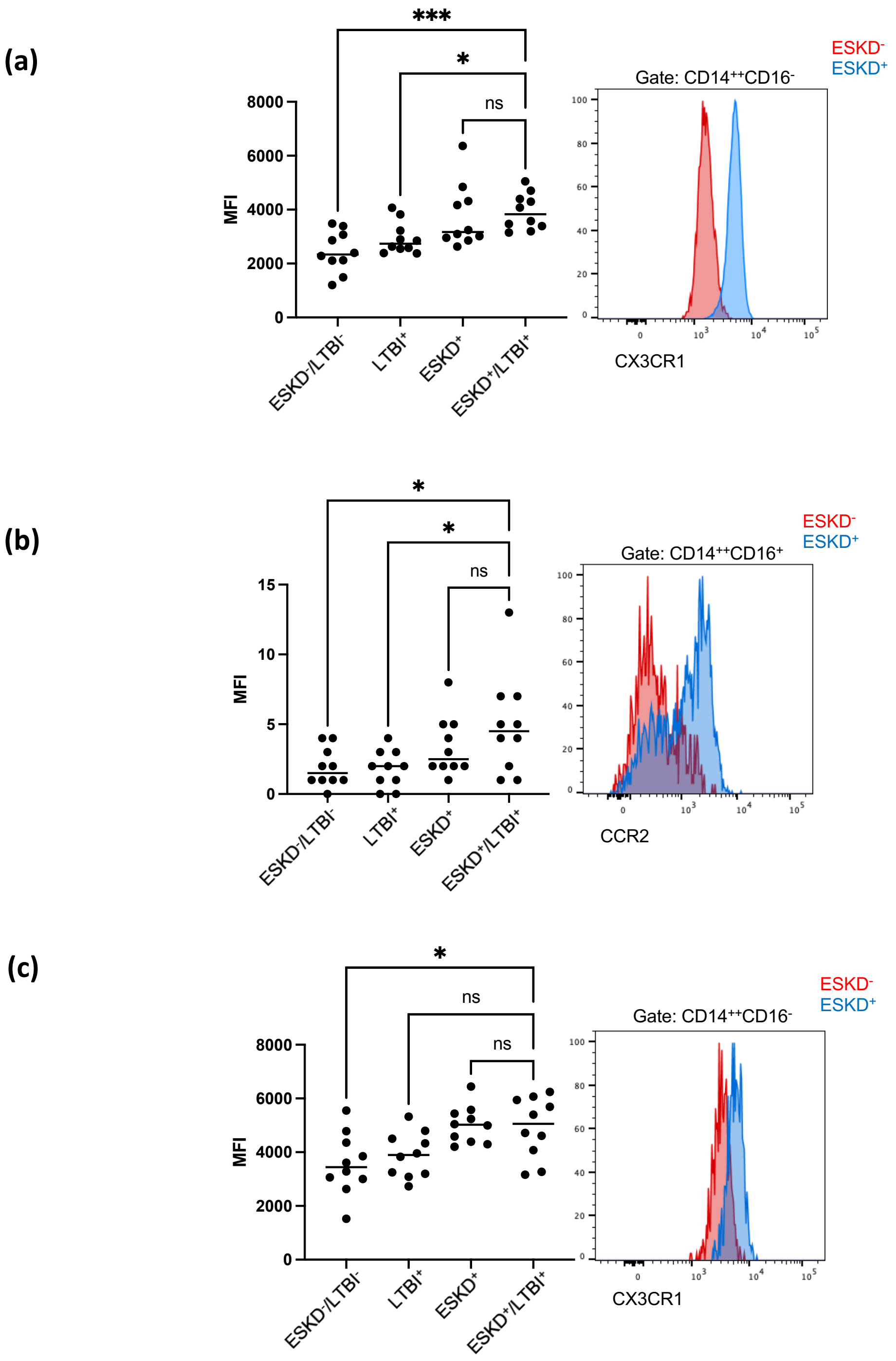

Supplementary figure 5. Monocyte staining of CXCR1^{+} and CCR2^{+} phenotypes. **(a)** Individual plots of MFI values for expression of CX3CR1^{+} on CD14^{++}CD16^{-} monocytes in ESKD^{-}/LTBI^{-} (*n* = 10), LTBI^{+}(*n* = 10), ESKD^{+}(*n* = 10) and ESKD^{+}/LTBI^{+}(*n* = 10). Gating compared MFI in ESKD^{-} (*n* = 20) to ESKD^{+} (*n* = 20). **(b)** Demonstrates expression of CCR2^{+} on CD14^{++}CD16^{+} monocytes while **(c)** demonstrates CX3CR1^{+} expression on CD14^{++}CD16^{-} monocytes.
